# Meditators’ Non-academic Definition of Mindfulness

**DOI:** 10.1007/s12671-022-01899-3

**Published:** 2022-05-24

**Authors:** David Alvear, Joaquim Soler, Ausiàs Cebolla

**Affiliations:** 1 Department of Developmental and Educational Psychology, HEFA I, University of the Basque Country UPV/EHU, Office 3B3.3. Avenida de Tolosa, 70. 20018, San Sebastian, Spain; 2 Servei1Servei de Psiquiatria, Hospital de La Santa Creu I Sant Pau, Barcelona, Spain; 3 Department of Psychiatry and Forensic Medicine, Universitat Autònoma de Barcelona (UAB), Barcelona, Spain; 4 Department of Personality, Assessment and Psychological Treatments, University of Valencia, Valencia, Spain

**Keywords:** Mindfulness, Lay theories, Qualitative research, Thematic analysis, Meditation

## Abstract

**Objectives:**

Mindfulness has been defined differently in academic scientific contexts and in Buddhist academic contexts. An under-studied area is that of lay (non-academic) theories of mindfulness. The goal of this article is to identify, organize, analyze in detail, and provide themes from the meditators’ definitions of mindfulness. Possible differences and similarities of the collected definitions of mindfulness with the scientific-academic definitions and with the academic-Buddhist definitions are also checked.

**Methods:**

A qualitative and inductive thematic analysis on the definitions of mindfulness offered by the participants was carried out.

**Results:**

The sample consisted of 326 meditators who offered a definition of mindfulness through an open question. Seven themes were identified: (1) mindfulness defined as attention/awareness; (2) mindfulness defined as a non-evaluative attitude; (3) mindfulness defined as strategy; (4) mindfulness defined from a theoretical analysis; (5) mindfulness defined as a psycho-affective-spiritual state; (6) mindfulness defined as personal development; and (7) lack of understanding of mindfulness. From these themes, it can be deduced that the definitions collected share more patterns of meaning with the scientific-academic definition of mindfulness than with the academic-Buddhist one.

**Conclusions:**

The findings of this study provide new insights into the complexity and heterogeneity of the definition of mindfulness. What has been discovered may indicate the complexity of the mindfulness construct itself.

**Supplementary Information:**

The online version contains supplementary material available at 10.1007/s12671-022-01899-3.

In recent decades, mindfulness has been defined in multiple ways in academic contexts, from a theoretical construct that can be measured (Baer et al., 2008; Brown & Ryan, 2003) to a type of meditation practice (Kabat-Zinn, 1994). One of the most accepted definitions in the academic field is the one that understands mindfulness as the ability to “pay attention in a particular way: on purpose, in the present moment, and non-judgmentally” (Kabat-Zinn, 1994). Other definitions suggested defining it as a trait. In that case, mindfulness can be understood as a relatively stable but trainable ability. Therefore, a fairly consensual model of trait mindfulness would have two components (Bishop et al., 2004): (a) self-regulation of attention so that it remains in the experience of the present moment and (b) the deliberate guidance of attention toward one’s own experience while approaching it with curiosity, openness, and an acceptance of bodily sensations, thoughts, and emotions.

The role of learning in mindfulness-based intervention (MBI) programs is the acquisition of mindfulness skills with the aim of achieving clinical, educational, social, or emotional outcomes (Crane et al., 2017). Different measuring instruments are used to understand the level of trait mindfulness and to evaluate the mindfulness competences acquired by participants (Baer, 2016; Soler et al., 2014). Although there are many advantages in using a psychometric approach to measure mindfulness (e.g., rapid application, known methodology, and statistical support) (Sauer et al., 2013), numerous criticisms have been associated with how problematic it can be to even define a construct as complex and with such a particular historical-spiritual journey as mindfulness, let alone try to measure it using self-evaluation instruments (Grossman, 2011; Grossman & Van Dam, 2011), or how the verbal nature of the questionnaires limits their capacity to assess non-conceptual aspects included in the construct (Tortella-Feliu et al., 2020). One of the most problematic issues is that the definition of mindfulness is different for each measuring instrument, due to the different theoretical models underlying each of them. As Grossman (2011) pointed out, one of the possible reasons is that even though an accepted definition, out of the dozens of existing scales, exists by consensus (Bishop et al., 2004), only the Philadelphia Mindfulness Scale (PHLMS) (Cardaciotto et al., 2008) is inspired by Bishop’s definition. What happens then is that studies that aim to analyze the correlations between mindfulness scales find that the mean correlation can range anywhere between 0.21 and 0.67 with an average value of 0.43 (Bergomi et al., 2013). One study even reported a total absence of correlations between the measurement scales (Thompson & Waltz, 2007). This could account for the fact that half of the MBIs failed to detect changes in self-reported mindfulness after ending the treatment (Visted et al., 2015).

This abundance of different mindfulness measurement scales does not seem to have reduced the problems of validity and reliability of the construct (Grossman, 2019; Tomlinson et al., 2018). In addition, quantitative approaches have been criticized and reported to be limited and biased in terms of the significant responses offered when analyzing multidimensional, complex terms with an experiential value (first-person experiences) such as mindfulness (Grossman, 2008, 2019). All of this has led to a gradual increase and a trend toward qualitative research that seeks to enrich the field of study of mindfulness (Frank et al., 2019). Thus, it is possible to offer a greater prominence to the subjectivity of the meditators or to the complex set of conditions determining whether and how the mindfulness training influences the attendees. (Frank et al., 2019; Garland & Gaylord, 2009; Tomlinson et al., 2018).

Regarding the problem of defining the construct, Grossman (2011) proposed another added difficulty, in which he pointed out that some authors are not sufficiently familiar with the theoretical and practical bases of the concept of mindfulness as presented in the Buddhist tradition. Yet another difficulty could be found when trying to unlink a single concept (mindfulness) from related concepts belonging to a conceptual framework of Buddhist origin at a theoretical level (Christopher et al., 2009) and, at the same time, carrying out this terminological decontextualization with different objectives. For this reason, some authors already recommended designing MBIs considering different types and dimensions of mindfulness, which would mean refining and integrating the complexity of the theoretical definition itself (Dorjee, 2010).

In the case of the Buddhist tradition, the purpose of cultivating mindfulness, different from what is proposed by the mindfulness known in the scientific field, is related to the search for liberation or awakening that can annihilate dissatisfaction (*dukkha*) (Sayadaw, 2016). Each Buddhist school, and even each author within each school, contributes certain nuances to the definition of mindfulness in numerous geographical locations and at different historical moments (Dreyfus, 2011; Dunne, 2015). In fact, the very translation of the term *sati* as mindfulness in the second half of the nineteenth century by T. W. Rhys Davis has never been clarified or justified at a philological level (Gethin, 2011).

Bhikkhu Bodhi (1993) defines mindfulness (the term *sati* in Pali) as “the mental ability to pay attention to physical or mental events that occur in the present moment.” Anālayo (2016) proposed a more complex and richer conceptualization of mindfulness, starting from an analysis based on Pali discourses from early Buddhism. Anālayo (2016) distinguished between different types of mindfulness (e.g., right mindfulness and wrong mindfulness; established mindfulness and lost mindfulness) and took a standard definition of mindfulness as “someone who is aware (mindful) and has the ability to remember what has been done or said a long time ago,” although he emphasized that this element of remembering would have to do with episodic memory. Levman (2017) gave the memory and recall factor implicit in the term *sati* a key importance, while Anālayo (2019a) indicated that in early Buddhism *sati* is not always understood as memory in terms of thought; in fact, he pointed out that in the context of *satipaṭṭhāna* meditation, the meaning “conscious attention to what is present” offers a better fit. Anālayo (2020) himself also differentiated between mindfulness and attention and considered them two different mental qualities, attention being a constantly present mental quality, while the mental quality of mindfulness would be intermittent, given that it would have to be activated and cultivated with practice. Based on the *Visuddhimagga* written by *Buddhaghosa* in the fifth century AC, Wallace (2008) pointed out that *sati* is characterized by properties such as “not floating,” “not getting lost,” “vigilant,” “being face to face with the object,” or “noticing strongly.” In reference to “not floating” (*apilāpana*), Anālayo (2019b) qualifies the term and provides a historical perspective on the change in meaning to which it was subjected. The debate surrounding the definition of mindfulness (*sati*) in Buddhist tradition remains open and with relatively clear socio-cultural influences (Dunne, 2015).

At present, the term mindfulness is popular in psychology, psychiatry, neuroscience, and education, and from there, it has spread first to other scientific fields and, later, to the rest of society via mass media, internet, non-fiction literature of an informative nature, and social networks (Van Dam et al., 2018). We could therefore justify that a large part of the Euro-American population has their own definition, a lay definition, of what they understand by mindfulness (Choi et al., 2021).

Lay theories are the informal theories that people (not academically specialized in a certain subject) use in their daily lives to explain different phenomena and their causes and consequences (Furnham, 1988; Levy et al., 2006). These lay theories serve as frames of reference that influence the processes of perception, interpretation of information, and prediction of events and, consequently, also people’s choices and behaviors (Furnham, 1988; Levy et al., 2005). Normally, lay theories are not based on scientific research, not even on a systematic study of the phenomenon in question, but are rather nourished by stereotypes and everyday beliefs (“everyone knows that ...”) (Ritter & Rietzschel, 2017). Lay theories can be found everywhere, implicit in everyday conversations between people, in non-scientific written publications (books, newspapers, web pages), in the media, on social networks, etc. When comparing lay theories with scientific theories, lay theories are not explicit, lack coherence and consistency, give more importance to content than to process, and tend to confuse correlation with causality (Furnham, 1988). For this reason, it is difficult to use the term lay “theory” as we understand it in science, as they are rather a dynamic group of implicit beliefs used systematically to explain and predict certain phenomena. There are several similar psychological terms such as implicit theories, lay theories, folk psychology, common-sense psychology, or mindset. In this article, they will all be represented by the term lay theory, *lay* in the sense of contrary to expert or scientific, every day, non-specialist beliefs of most people on a subject, in this case mindfulness. By *theory*, we understand that there is a certain interconnection between these ideas about mindfulness.

There is a wide body of literature that refers to lay theories, as opposed to academic ones, on various psychological phenomena (Furnham, 1988). Regarding mindfulness, lay beliefs of attention and awareness have been previously addressed to understand their implication in the workplace (Kong & Jolly, 2019). However, the debate on the suitability of the dichotomy between lay and scientific expertise is open. There are authors who, in the case of mindfulness, classify it as problematic; they consider it highly questionable that expertise (in contrast to lay perspectives) evolves only through academic engagement (Grossman, 2019).

The definition that each person has of mindfulness can have a direct impact on their own mindfulness practice, on the level of adherence to the practice, and on the results from the practice itself. Subjectivity and the preponderance of first-person experiences have been a prominent element in mindfulness meditation. Each practitioner experiences different variables from aspects that are ineffable or difficult to label through language. For example, the practice of mindfulness can modify factors such as the subjective experience of time (Droit-Volet et al., 2018). The meditating subject is immersed in a meditative tradition and in a culture that sustains and contextualizes the subjectivity of the experience itself (Reddy & Roy, 2019). Since Varela & Shear (1999) promoted first-person approaches in the contemplative sciences, several authors have stressed the need to integrate first-person and third-person research in this type of science (Hadash & Bernstein, 2019).

This interest in first-person experiences has led to qualitative methodology studies that prioritize the narrative-experiential basis and the phenomenological aspects of the meditator. Therefore, this study can be considered an initial qualitative approach with the aim of understanding and analyzing, in a thematic way, the experimental, semantic, and phenomenological richness of the definitions of mindfulness in Spanish-speaking meditators.

## Method

### Participants

A total of 326 participants completed the questionnaire, of which 325 (99.7%) responded to the open-ended question to be analyzed. Responses that did not adhere to the open-ended question were not detected, so all 325 definitions were analyzed. The mean age of the sample was 50.2 years (SD = 10.6, range = 25 to 74) and 72.7% were women. All participants had meditated or practiced mindfulness; 23.6% currently did not meditate regularly, 13.8% had been meditating regularly for less than a year, 20.2% had been meditating between 1 and 3 years, 26.1% had been meditating regularly for 4 to 10 years, and 15.6% had been meditating regularly for more than 10 years. A total of 63.7% of the sample that meditated regularly indicated that mindfulness was the type of meditation that best suited their practice. Regarding the type of meditative practice, 63.7% practiced mindfulness, 4.6% practiced Zen, 4.3% practiced Vipassana, 2.8% practiced a combination of mindfulness with another type of meditation, 2.5% practiced Yoga, 1.8% practiced Christian meditation or prayer, 1.5% practiced Buddhist meditation (generic), and 12.9% practiced another type of meditation.

### Procedure

An online questionnaire was developed on a commercial platform (https://docs.google.com/) and distributed through various websites and social networks in Spanish on mindfulness and meditation. The participants were informed about the research and invited to participate in the study to “understand your opinion regarding mindfulness and meditation.” The date was processed anonymously and in accordance with the Spanish Data Protection Law. The link was available for 3 weeks (from April 15, 2020, to May 6, 2020).

### Measures

The questionnaire in its extended version contained contextual data related to meditation practice and sociodemographic variables, as well as other types of open-ended questions on contemplative sciences. To achieve the objectives proposed in this study, we analyzed the following open-ended question: “Using your own words: How would you define mindfulness?” The answer did not have any character limitation and each participant could develop the answer freely. We have assumed that the responses to this open-ended question reflect the most important definition, concepts, and narratives regarding what participants understand by mindfulness.

### Data Analyses

This research was carried out following the interpretive paradigm, focused on the study of the meanings of human actions and social life, that is, the interpretation that social actors make of their “reality,” and emphasizes the researcher’s process of understanding to try to interpret the meaning of their actions (Daly, 2007). According to Willis (2007), the interpretive paradigm is in favor of qualitative methods, given that they can offer a more profound approach when it comes to understanding how human beings interpret the world around them.

In the present work, a qualitative analysis with a particularly inductive character was chosen to search for the data’s underlying categories. A bottom-up approach to the data analysis was used, with the authors primarily using the data obtained from the participants’ responses. To analyze the answers to the open-ended question, we used the method employed in thematic analysis. This method makes it possible to identify, organize, analyze in detail, and provide patterns or themes from a careful reading and rereading of the collected information, and thus infer results that promote the adequate understanding/interpretation of the study’s phenomenon (Braun & Clarke, 2006). In this way, we followed a systematic process to find response patterns or themes embedded in the narrative of the responses. The data analysis was carried out by two members of the team, scientific psychologists who are experts in mindfulness and/or qualitative methods. The six phases of analysis that were carried out are the following (Braun & Clarke, 2006):

1. Data familiarization: reading and rereading all the answers to the open-ended question and taking notes of the general ideas
2. Initial code creation: all relevant words, phrases, or paragraphs (units of meaning) that were related to the research question were extracted and coded. This code was a brief labeling that captured the essence of the unit of meaning. The codes were then organized in a specific code table
3. Theme search: codes compilation on possible themes and gathering of all relevant data for each theme
4. Theme review: The themes were reviewed and organized in a coherent pattern. A consistent pattern includes internal homogeneity (e.g., codes are conceptually integrated in each theme) and external homogeneity (e.g., there is a clear distinction between themes). The team subsequently re-examined all definitions as a whole to ensure that all relevant units of meaning were captured by one of the themes
5. Theme definition and naming: constant analysis to adjust the details of each theme and creation of clear definitions and names for each theme
6. Report: Finally, an academic report on the analysis was written with detailed information on each theme.

Reliability was obtained with two evaluations. The first was based on expert judgment, following reflection, discussion, and criticism (Ahuvia, 2001) with an international specialist. The second was obtained through agreement between the coders and was the product of systematic reflections to define and establish the codes (subcodes) and categories (subcategories). Validity was obtained during this agreement between the coders, in which an external coder assigned the same analysis codes in the same categories according to the coding carried out by the research team (Vaismoradi et al., 2013).

## Results

The thematic analysis carried out based on the 325 definitions of mindfulness derived from the open-ended question “Using your own words: How would you define mindfulness?” offered seven main themes: attention/awareness, non-evaluative attitude, strategy, theoretical analysis, psycho-affective-spiritual state, personal development, and lack of understanding (see Table 1). Each theme is described below.

**Table 1:** Summary of themes and subthemes obtained regarding the lay definition of mindfulness

*Themes*	Subthemes
*Mindfulness defined as attention/awareness*	Attention to the present Full awareness “*Atención plena*” (synonymous definition) Presence Attention when doing Attention with intention Being aware/noticing Attention to different phenomena (breath, body, thoughts, or emotions) Observing Remembering Concentration
*Mindfulness defined as a non-evaluative attitude*	Non-judgmental attitude Acceptance and equanimity attitude Curious attitude Kind and/or compassionate attitude
*Mindfulness defined as strategy*	Strategy to regulate emotions or the mind Strategy to regulate attention
*Mindfulness defined from a theoretical analysis*	General theoretical analysis Technical theoretical analysis
*Mindfulness defined as a psycho-affective-spiritual state*	A state of well-being A psychological state A spiritual state
*Mindfulness defined as personal development*	Lifestyle Self-knowledge Connection with oneself Connection with the environment
*Lack of understanding of mindfulness*	Total or partial ignorance

### Mindfulness defined as attention/awareness

In this theme, the main and transversal element is that of attention. A large majority of the sample defined mindfulness as attention, especially as “paying attention to the present moment” (*n* = 191). Understanding that the subthemes are essentially themes within a theme, ten subthemes were identified in the theme *mindfulness defined as attention/awareness*: attention to the present, full attention, presence, attention when doing, attention with intention, being aware/noticing, attention to different phenomena, observing, remembering, and concentration.

Some of the participants expressed their attention to the present moment in a terminologically close way but with qualitative nuances that we consider important to highlight. In this theme, we wanted to highlight that what may appear to be concepts that are linguistically close (e.g., “attention to the present” vs. “full attention”), at a semantic and even axiological level, have differential characteristics that justify that we have created numerous subthemes. We are aware that when doing thematic analysis, the subthemes generally should be used sparingly, only when there is one particular element of a theme that has a particular focus, is notable, and/or is particularly important for the research question. However, we believe that the idiosyncrasy, breadth, and numerous nuances in the more than 300 definitions covered by this theme itself lead to a distinction in a large number of subthemes. Thus, there were definitions that highlighted the aspect of a spatio-temporal setting (*n* = 43) with expressions such as “here and now,” and others that gave it a different nuance by using “presence” (*n* = 12). Quite a few people chose to define mindfulness with a synonym such as the term “*atención plena* [Spanish for mindfulness or full awareness]” (*n* = 41). Some participants were more specific and spoke of being focused and aware when doing (something) (*n* = 17), and one participant pointed it out as “being in what you do.” Other members of the sample went a little further into detail and defined mindfulness directly as “concentration” (*n* = 10), “concentrating on the present.”

Definitions were also detected that, without explicitly using the term attention, indicated verbs with a visual sensory basis such as observe, see, or look (*n* = 10) (e.g., “observe what is there”). Some indicated an attention with an intention (*n* = 13), an attention that implies a certain will. In this regard, one person wrote, “paying attention deliberately,” and another indicated, “paying attention with the intention to do so.”

Multiple definitions were found that referred to attention paid to different phenomena such as “attention to breathing” (*n* = 9), “attention to thoughts or mind” (*n* = 14), “attention to emotions or feelings” (*n* = 14), or “attention to the body” (*n* = 21). One person defined it as “becoming aware of the sensations of your body when you notice them.”

It is important to highlight that part of the sample, instead of (or in addition to) defining mindfulness as attention, preferred to use the term “being aware” (*ser consciente* in Spanish) (*n* = 63) or “noticing” (*darse cuenta* in Spanish) (*n* = 9), which, as we understand it, offers a phenomenologically and epistemologically differential nuance of how one relates to one’s experience. One person spoke of “simply being aware of what arises,” and another pointed it out as “becoming aware of everything that is happening at all times.”

Finally, there was a way to define mindfulness from an attentional aspect but with an evident memory weight; it is what we have called “remembering” (*n* = 2), and one subject defined it as “remembering to pay attention.”

### Mindfulness defined as a non-evaluative attitude

Part of the definitions of mindfulness implied an attitude, a way of coping, a concrete way of relating to what is experienced. We called this theme non-evaluative attitude. Four subthemes were detected within this theme: non-judgmental attitude, acceptance and equanimity attitude, curious attitude, and kind and/or compassionate attitude. The “non-judgmental” attitude stands out (*n* = 47) within this non-evaluative attitude theme. This type of attitude implies approaching what is experienced without judgment, or, at least, with as little evaluation as possible.

The acceptance or equanimity attitude (*n* = 34) implies another way, perhaps more passive and receptive, of carrying out this non-evaluative attitude, as one person pointed out “accepting what comes.” On the other hand, the curious attitude (*n* = 10) could indicate a more active and inquiring approach to what is experienced, as one participant reported “paying attention with curious interest.” Finally, the attitude of kindness and/or compassion was also indicated with a more affective basis (*n* = 22). One participant said that mindfulness meant “bringing attention to the present in a gentle way.”

### Mindfulness defined as strategy

Another different way that the participants chose to define mindfulness was to understand it as a strategy. In this case, the meaning of mindfulness was no longer considered a direct conceptual definition such as attention, but rather defined as something instrumental, as a means to an end. Two subthemes were detected: strategy to regulate, manage, or control emotions or the mind, and strategy to regulate attention.

We observed how part of the sample defined mindfulness as a regulation strategy or as a tool for emotional or mental control, although the approach to what type of regulation strategy involves mindfulness is diverse. Some did propose a clear definition of mindfulness as an element of emotional regulation (*n* = 7), “it is a set of skills for managing the mind and emotions,” while other definitions suggested that mindfulness regulated emotions by being a thought-avoidance strategy or a “not thinking” strategy (*n* = 6), “it serves to stop automatic thoughts” or “it seeks to stop the mental chatter.” Something terminologically close came from the one that understood it as a calm or, even, an “emptying the mind” (*n* = 6); one person defined it as “a way of quieting the mind.” Other proposals in these lines of emotional/mental regulation would be those that understood mindfulness as “stopping” or “a way to stop” (*n* = 6), and as a strategy to let go and/or release (*n* = 6). One participant expressed it as “free your mind,” and another suggested that it involved “constantly letting go from attention the objects that appear in one’s consciousness.” Some participants understood mindfulness as a strategy based on disidentification (*n* = 6). One said that mindfulness was “(paying attention to the mind) without identifying with the contents.” And others understood it as an aid to act (*n* = 4), “a state that predisposes one to action,” or even for “mind control” (*n* = 2).

The second subtheme detected within this theme was mindfulness understood as a strategy to regulate attention (*n* = 10). Here, the participants highlighted the capacity offered by the practice of mindfulness to not direct attention to the past or to the future and to focus attention on the present, offering special emphasis on the process itself, “being really in what I am and, when I’m leaving, becoming aware that I have gone.” Another one added importance to the ability to direct attention at will, “the ability to direct attention to what is wanted.”

### Mindfulness defined from a theoretical analysis

There were participants who chose to define mindfulness by carrying out a theoretical analysis, reflecting and providing a conceptual description of the term. In this theme, we detected two subthemes: a general theoretical analysis that each person offers from their meditation and contextual experience, and a more defined theoretical analysis in which mindfulness is analyzed from a more technical and concrete perspective.

In the subtheme of the general theoretical analysis (*n* = 11), the diversity in the approach to what mindfulness is can be highlighted. There are participants who defined it as a way to simplify or make the practice of meditation more pragmatic, one pointed out that “it is a good simplification,” and another said that “it is a pragmatic turn to traditional meditation.” Others were more critical and spoke of mindfulness as “a trend,” as “a more superficial way than the original forms of meditation (yoga, Zen).” One participant even defined it as “decaffeinated Buddhism for western companies.”

Regarding the other subtheme, referring to the theoretical analysis in which mindfulness is defined from a more technical and concrete perspective (*n* = 44), participants came to catalog it into five different categories. Some thought that mindfulness “is a type of meditation,” others reported that it is a training or a practice, with one saying that it is “the training of attention,” whereas others saw it more as a process or path, “it seems like a path.” There were people who related it to “the wisdom that is implied” in the mere fact of practicing mindfulness, and others indicated that it was more of a type of yoga, “it is a yoga of the mind.”

### Mindfulness defined as a psycho-affective-spiritual state

Some participants indicated that they understood mindfulness as a state. Among the people who defined mindfulness as a state, the answers were grouped into three subthemes: those who understood it as a state of well-being, as a psychological state, and as a spiritual state. Among those who referred to mindfulness as a state of well-being (*n* = 39), the answers were diverse when it came to specifying that state, some spoke of “general well-being,” while others reported positive emotional states related to activation such as “positivity,” “energy,” or “strength”, and others indicated positive states more related to deactivation such as “calm,” “serenity,” “relaxation,” or “a state of tranquility.” Other people connected mindfulness with a more generic and affectively neutral psychological state (*n* = 3), with one participant pointing out that it is “a state of mind.” In the third subtheme, some participants explained mindfulness as a state of spiritual nature (*n* = 11), some spoke of “return to peace” or only “peace,” while others referred to it as “Being” or “Stillness.”

### Mindfulness defined as personal development

Definitions were detected in which the participants understood mindfulness as an integrated element in their lives and that it implied support in their personal development. In this broad theme, we identified four subthemes: mindfulness as a lifestyle, as self-knowledge, as an element of connection with oneself, or as an element of connection with the environment.

There were participants who understood mindfulness not only as a practice or a state, but also as a “lifestyle” or a “life philosophy” (*n* = 14), something that involved everyday life and a vision for the future. In this regard, answers were found to express that “more than a meditation it is a way of life” or that mindfulness “is a way of being in life.” Other people related mindfulness to “self-knowledge” (*n* = 7), with one participant pointing out that it is “a way of self-exploration,” and another writing that “it is an indispensable practice for self-knowledge.”

Some participants identified mindfulness as an important element of “connection with oneself” (*n* = 24), and illustrated it in different ways, “it is an encounter with myself,” or “it is the ability to go within yourself,” or even “it is the ability to pay attention to what is happening in your inner world.” However, others gave weight not only to the connection with oneself, but also to the potential of mindfulness for the “connection with the environment” (*n* = 4), with one participant pointing out that mindfulness implied “the awareness of your whole being and what surrounds you.”

### Lack of understanding of mindfulness

A group of people showed total or partial ignorance (*n* = 10) regarding the meaning of mindfulness and, obviously, did not offer a definition. Some were brief in their response, pointing out that “I don’t know what mindfulness is, I don’t understand it,” and others did not dare to define it because they did not have the necessary knowledge, “I am still too new to define it properly.”

## Discussion

The present study collected seven descriptive themes that revealed the different types of definitions surrounding the concept of mindfulness. These themes were identified through a systematized analysis of the qualitative data obtained from the definition of mindfulness reported by each of the participants. The results of this study revealed the complexity of the mindfulness construct (Grossman & Van Dam, 2011), and the even greater linguistic and semantic dispersion and diversity that is found in non-academic definitions, compared to academic ones. At the same time, the strong socio-cultural influence on the definition itself was verified and we can speculate that there are many reasons why these definitions may vary (i.e., personality or learning history) (Dunne, 2015).

As presented in the results, seven themes were conceptualized that represent the definitions obtained from the data. As expected, the subject of *mindfulness as attention/awareness* stood out at a quantitative and qualitative level; most of the participants used some unit of meaning that was articulated from attention or awareness to define mindfulness, in such a way that conceptual similarities were found with the scientific-academic definition of mindfulness, where the importance offered to attention and awareness is decisive (Bishop et al., 2004; Kabat-Zinn, 1994). Another similarity observed was the importance that the theme of *mindfulness as a non-evaluative attitude* took in the collected definitions, coinciding with the psychometric weight of factors such as acceptance or non-judgment in scientific-academic definitions (Baer et al., 2008; Bishop et al., 2004). However, participants’ definitions that resembled those proposed by Buddhist academics were limited. In this regard, mindfulness (*sati*) understood as remembering, important in tradition, was reported only by two people, and mindfulness integrated in an ethical-moral aspect was not pointed out by anyone. This may indicate that, at a pedagogical level, the dissemination of scientific mindfulness has permeated the public in a more evident way than the dissemination of Buddhist mindfulness. On the other hand, the aspects debated at a scientific-academic level, such as the differences between paying attention and awareness (Travis et al., 2017) or the differences between awareness and concentration (Mikulas, 2015), were also included in the collected definitions, although with varied terminological connotations and not always coinciding with the academic ones. In non-academic definitions, the linguistic complexity and the variety of terms were greater than in the academic definitions, detecting up to ten subthemes referring to the subject of attention/awareness. In general, we can say that, in conceptual terms, the lay or common-sense definitions were quite aligned with the scientific-academic definitions and the construct of mindfulness itself, although they differed in the importance they gave to each dimension (attention to the present moment vs. acceptance/non-judgment), with the importance given to attention/awareness to the present being greater in the collected definitions. This study can also confirm that the definitions that come from the Buddhist school have little conceptual weight in the definitions collected.

The theme that includes mindfulness as a strategy coincides with the mechanisms of action proposed by the literature to explain how mindfulness meditation works (Hölzel et al., 2011), especially the mechanisms that refer to the regulation of attention and the regulation of emotion. Along with this, some definitions were found that were not very compatible with scientific-academic definitions. Some participants defined mindfulness as flowing or being completely concentrated, which seems to be referring to the construct of flow (Csikszentmihalyi, 1990), rather than to that of mindfulness. Specifically, two categories detected within the subject of mindfulness understood as a strategy of emotional and/or mental regulation, such as thought-avoidance or “not thinking” strategies and objective activity, seemed highly incompatible with the scientific definition of mindfulness. However, while these characteristics of mindfulness were not necessarily reflected in the scientific literature, they were sometimes described as such by leading mindfulness practitioners and trainers (Knuf, 2019).

Some definitions were inconclusive or not very explicit, and limited themselves to defining mindfulness with a synonym (*“atención plena [Spanish translation for mindfulness or full awareness]”*). An explanation could be that having carried out the study in Spanish, and having used an Anglicism (mindfulness) when asking the open-ended question, made it possible for some participants to be satisfied with the mere translation of the term from English to Spanish.

The data also highlights the wealth of perspectives with which mindfulness was understood in the population; although most opt for a definition related to attention/awareness, some referred to mindfulness as a strategy to achieve an end, such as a psychological or spiritual state, or as a path to self-knowledge, of connection with oneself, or, even, as a life philosophy. Some of these issues were not covered in scientific-academic definitions or in Buddhist ones. In the case of collected definitions, the influence of popular or self-help literature on mindfulness, or on meditation in general, may be a causal factor to consider. Criticism surrounding mindfulness meditation was also collected, several of them implying that scientific mindfulness was a trend, indicating its superficial and utilitarian character, compared to what the Buddhist contemplative tradition proposed through the integration of ethical and spiritual aspects. This line of criticism has already appeared in the scientific literature (Grossman & Van Dam, 2011).

Derived from the thematic analysis of the definitions, it could be hypothesized that the concept of attention was the linguistic reference that stood out. Furthermore, due to its difference in meaning with respect to the rest of the themes, the theme referring to *people who do not know mindfulness* stood out. In this study (with a sample that spoke Spanish), less than 5% (4.7%) of the participants did not understand mindfulness. Thus, it could be hypothesized that the vast majority of meditators, no matter what meditation they practiced, were able to offer a definition of mindfulness.

### Limitations and Future Research

What has been discovered with this study must be considered under the prism of the different existing limitations. Given that the answers to the open-ended question were anonymous and in writing, we were not able to do any follow-up to clarify ambiguous questions or to validate conclusions with the participants. In this line, there is a potential coverage and self-selection bias related to the online questionnaire and its distribution on web pages and social networks related to the field of mindfulness and meditation. Also, the data collection of the questionnaire was carried out in the midst of the global Covid-19 pandemic, and this very special contextual situation could have modified the responses.

The sample that responded to the questionnaire is culturally and linguistically limited, since it was only possible to answer the questionnaire in Spanish. Furthermore, the digital divide and limited ability with new technologies generate a cut-off point when accessing the questionnaire, so the possible answers have been limited only to the population with access and sufficient capacity to use new technologies and the Internet. Also, the sample of the study consists of 76.4% regular meditation practitioners, out of which almost 15.6% have been practicing mindfulness regularly for more than 10 years. From this perspective, it can be difficult to understand such a cohort as “lay people,” as probably many of them have more extensive mindfulness experience than many academics. Therefore, it may be risky to call the results obtained a “lay definition.”

The data collection regarding the lay definition of mindfulness was carried out only through an open question in a questionnaire; this contribution may be limited if we want to understand the phenomenon in all its complexity. The data obtained in focus groups or in a semi-structured interview with each participant would provide a broader and deeper vision of the studied phenomenon. Finally, in the early phases of the study, no steps were taken to discriminate between academic and non-academic meditators. Thus, it is not possible to reliably know if the sample is offering non-academic definitions.

Looking ahead to future research, it seems convenient to continue studying the non-academic definition of mindfulness with the aim of better understanding both the lexicon and the educational methods in the teaching-learning processes of mindfulness in the different interventions.

The present study provides new information on the complexity and heterogeneity of the non-academic definition of mindfulness, and the differences and similarities between non-academic definitions compared to academic scientific definitions and Buddhist academic ones. This study points to the diversity of mindsets when it comes to understanding mindfulness and highlights the need for additional research to better comprehend what the population understands by mindfulness.

## Supplementary Information

Below is the link to the electronic supplementary material.

Supplementary file1 (PDF 13 KB)
