# Supplementary material for: Meditators’ Non-academic Definition of Mindfulness
Source: Mindfulness (N Y). 2022 May 24;13(6):1544–54. doi: 10.1007/s12671-022-01899-3 (PMC9127491; doi:10.1007/s12671-022-01899-3)
Supplement: Supplementary file 1 — Supplementary file1 (PDF 13 KB) [file 12671_2022_1899_MOESM1_ESM.pdf]

**Supplementary Materials:**

We have publicly published the raw data and the structure of the categorization system carried out to obtain the themes and sub-themes in the following link:

https://osf.io/7yrwd/?view_only=ac470e10ecc343d7981bf2ea464dfe16
